# Supplementary material for: FEP Encapsulated Crack-Based Sensor for Measurement in Moisture-Laden Environment
Source: Materials (Basel). 2019 May 9;12(9):1516. doi: 10.3390/ma12091516 (PMC6539873; doi:10.3390/ma12091516)
Supplement: Supplementary file 1 [file materials-12-01516-s001.pdf]

materials

MDPI

Supplementary Materials

FEP Encapsulated Crack-Based Sensor for Measurement in Moisture-Laden Environment

Minho Kim [1,†], Hyesu Choi [1,†], Taewi Kim [1], Insic Hong [1], Yeonwook Roh [1], Jieun Park [1], SungChul Seo [2], Seungyong Han [1,*], Je-sung Koh [1,*] and Daeshik Kang [1,*]

[1] Department of Mechanical Engineering, Ajou University, Suwon 16499, Korea; hgh7706@ajou.ac.kr (M.K.); michelle0407@ajou.ac.kr (H.C.); rsb5287@gmail.com (T.K.); his3080@gmail.com (I.H.); ckj2065@naver.com (Y.R.); n9near@ajou.ac.kr (J.P.)

[2] Department of Health, Environmental and safety, EulJi University, Seongnam 13135, Korea; seo@eulji.ac.kr

† These authors contributed equally to this work

* Correspondence: sy84han@ajou.ac.kr (S.H.); jskoh@ajou.ac.kr (J.K.); dskang@ajou.ac.kr (D.K.)

Keywords: strain sensor; thin metal film; spider inspired; crack; encapsulation

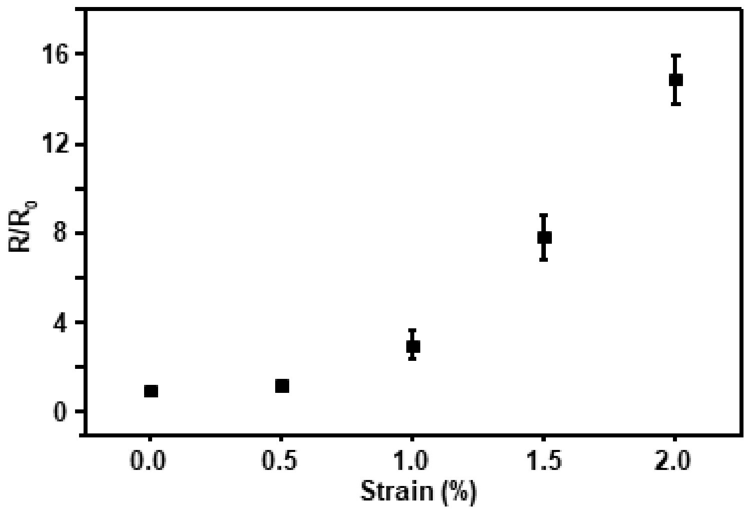

Figure S1. Reproducibility of the FEP encapsulated sensor.

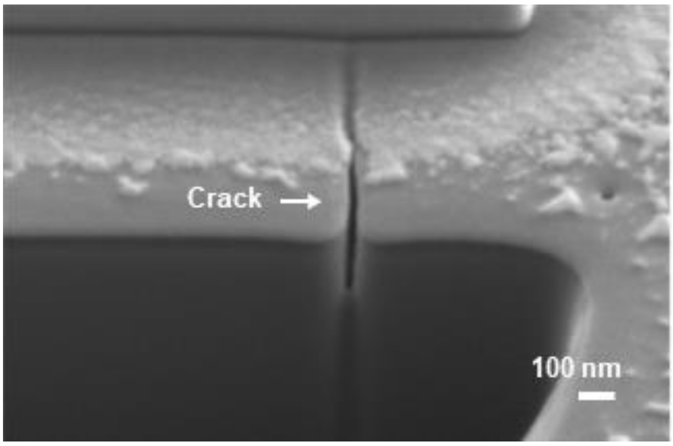

Figure S2. FIB Image of a crack on the sensor's surface.

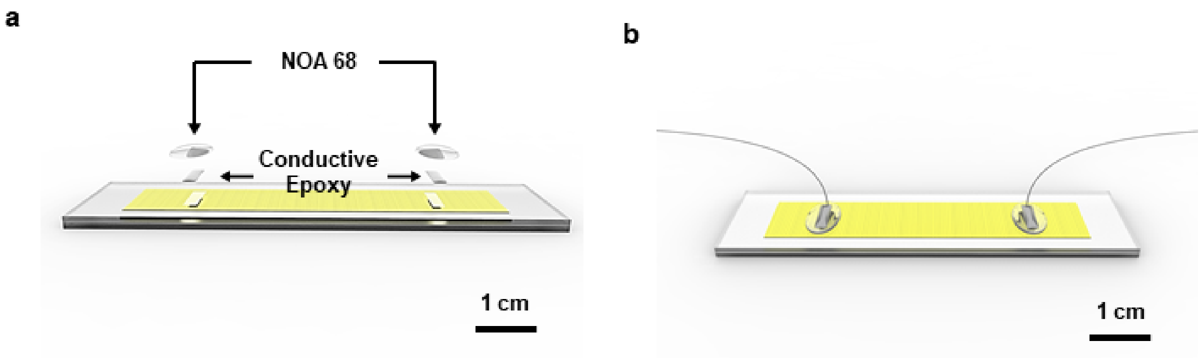

Figure S3. (a) Schematic illustration of wire-connecting method. (b) Schematic illustration of wire-connected FEP encapsulated sensor.

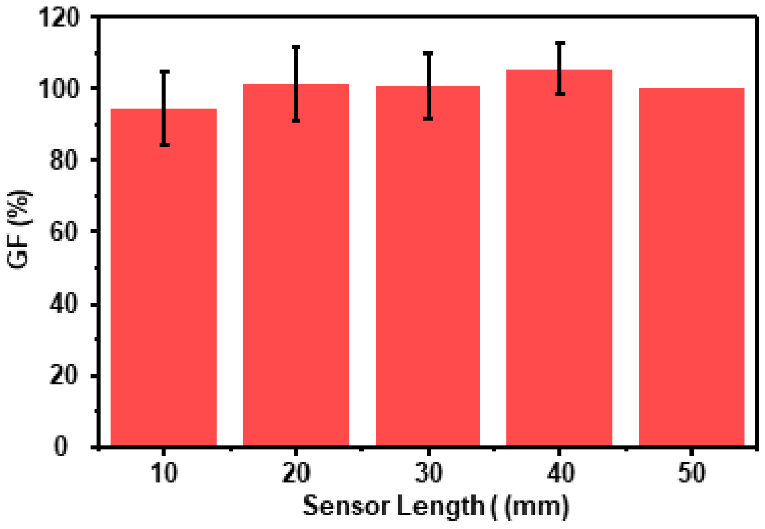

Figure S4. Gauge Factor variation of sensors with different length. (Percentage calculated from a sample with 50 mm length).

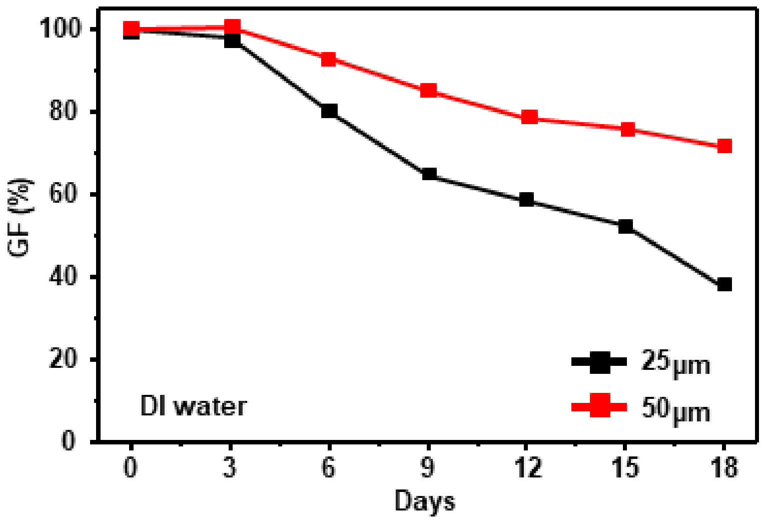

Figure S5. Gauge Factor variation of sensors encapsulated by 25 μm FEP films and 50 μm FEP films that have been in water for 18 days.

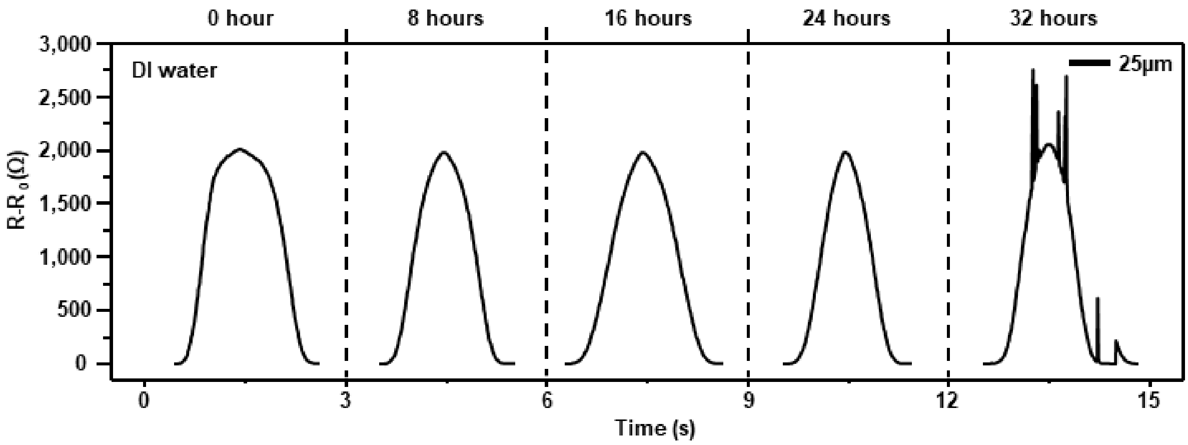

Figure S6. Resistance variation of bare crack sensor that have been in water for 32 h.

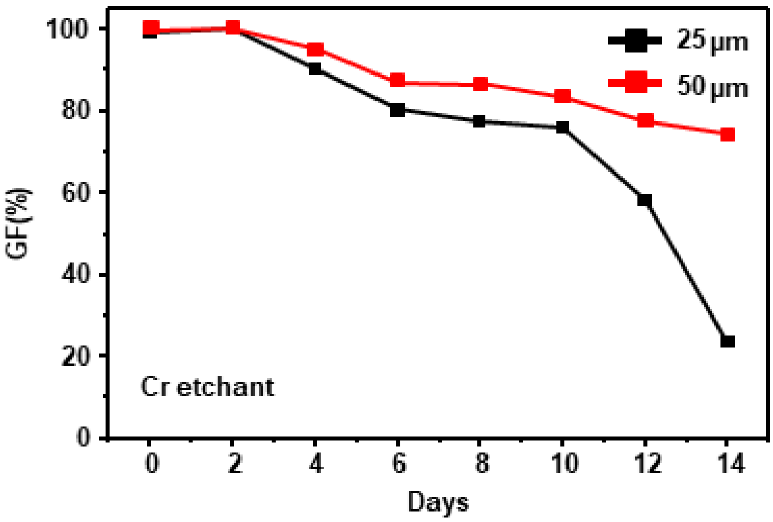

Figure S7. Gauge Factor variation of sensors encapsulated by 25 μm FEP films and 50 μm FEP films that have been in chromium etchant for 14 days.

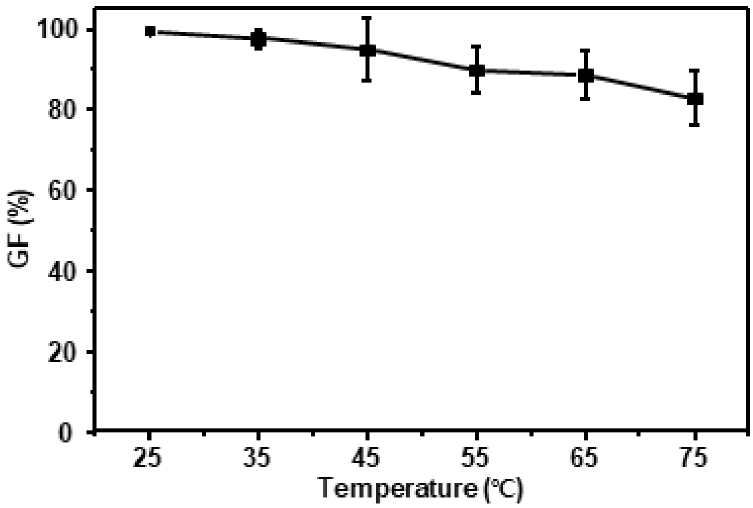

Figure 8. Gauge Factor variation of the sensor encapsulated by 25 μm FEP films from 25 °C to 75 °C.

Table S1. Sample size, Mean and Standard deviation of the resistance variation data from finger motion test in Figure 4c.

	Sample size	Mean	Standard Deviation
air	10	0.166465	0.006656
water	10	0.147988	0.006264

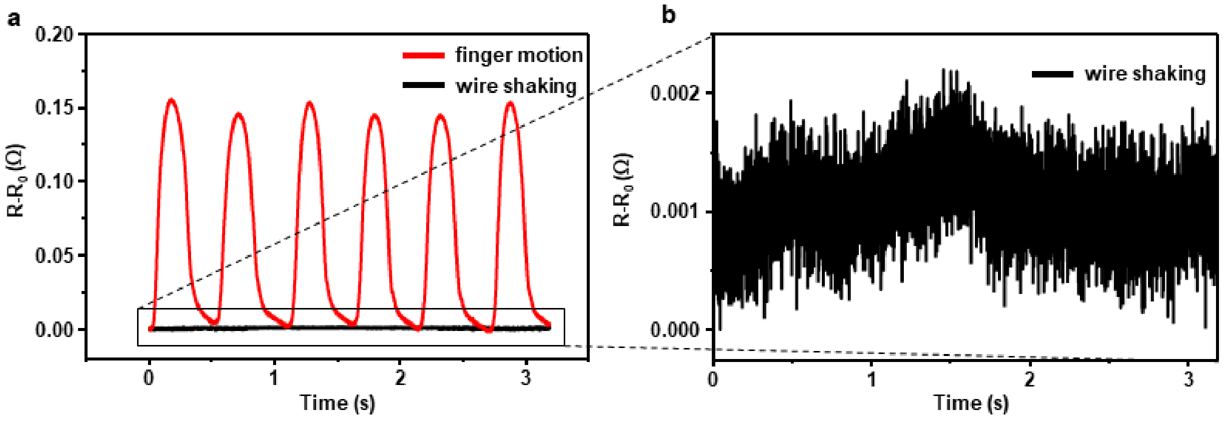

Figure S9. (a) Resistance variation of the sensor encapsulated with the 25 μm FEP film during the finger motion test. (b) Shows a small-scale plot for the wire shaking.

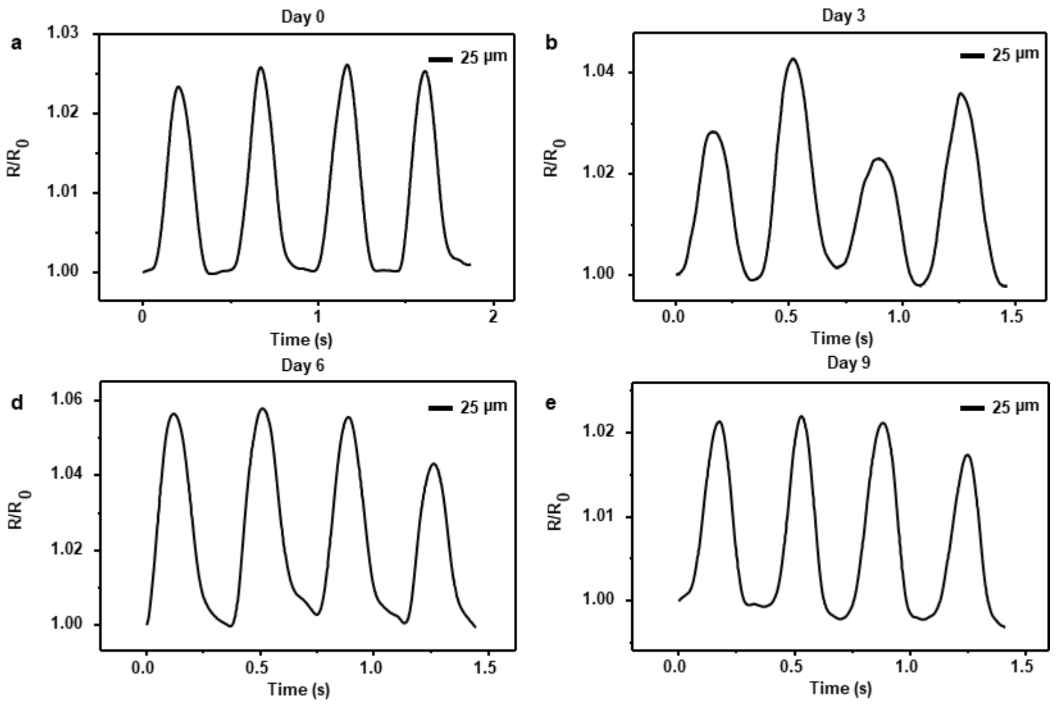

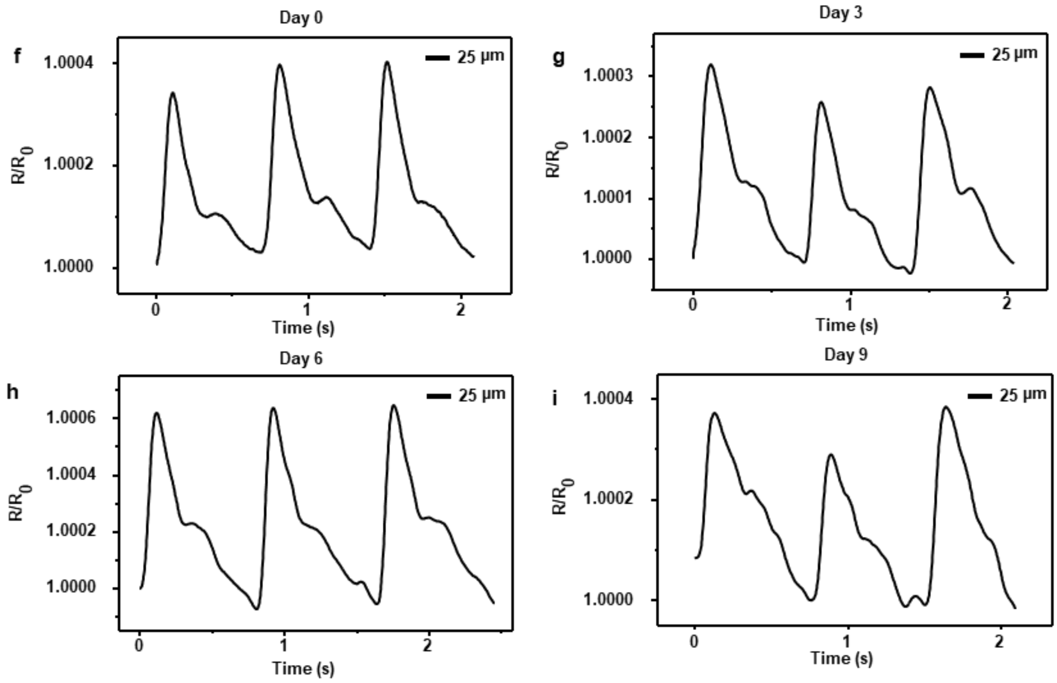

Figure S10. (a), (b), (c), (d) Measurement of finger's motions using the sensor encapsulated with the 25 μm FEP film that have been soaked in water for 9 days. (f), (g), (h), (i) Measurement of pulse rate using the sensor encapsulated with the 25 μm FEP film that have been soaked in water for 9 days.
